# Supplementary figures and images for: Sediment source and dose influence the larval performance of the threatened coral Orbicella faveolata
Source: PLoS One. 2024 Jun 26;19(6):e0292474. doi: 10.1371/journal.pone.0292474 (PMC11207144; doi:10.1371/journal.pone.0292474)

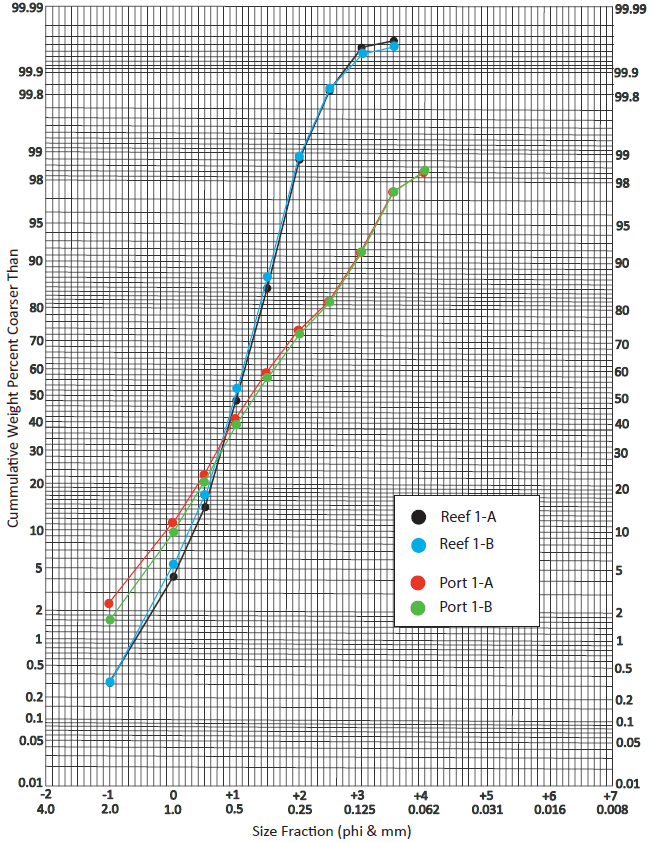

Supplement: S1 Fig — Two replicates per sediment type (Port versus Reef) were used. (DOCX) [file pone.0292474.s001.docx]

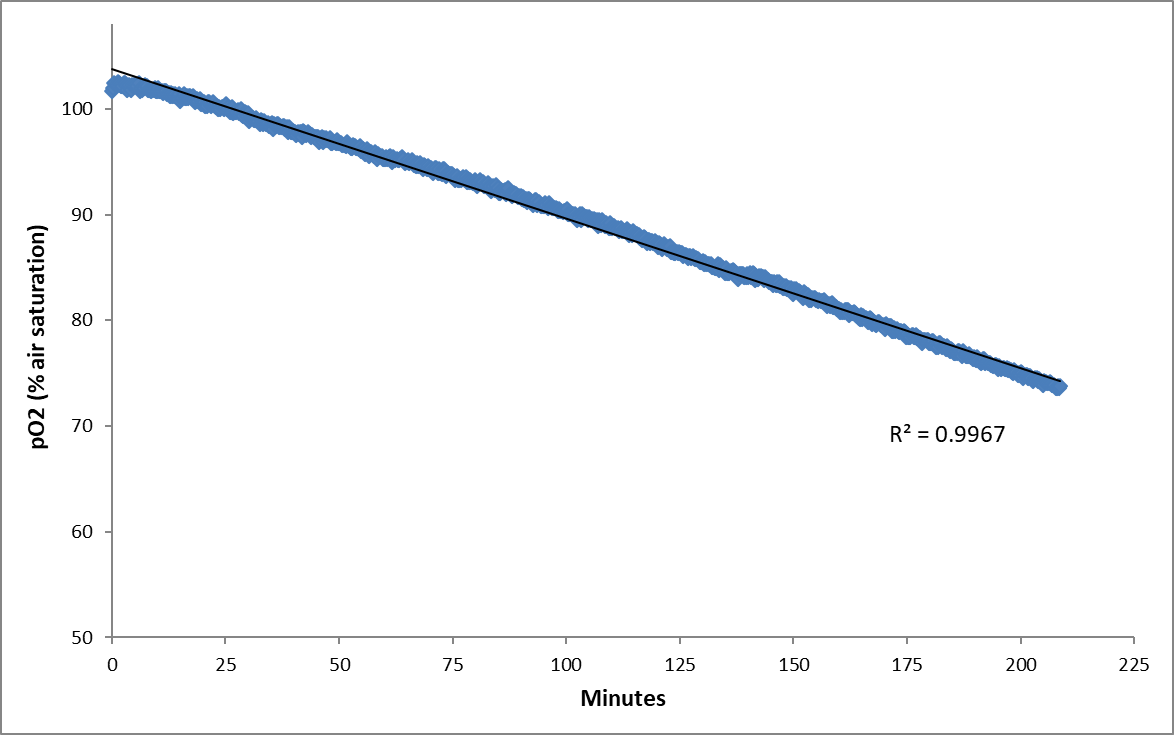

Supplement: S2 Fig — The R2 value is given for the linear trend. The first 10–20% (pO2 in % air saturation) linear decreases in oxygen were used to calculate respiration rates per larva. Any portion of the slope that dipped below 70% air saturation was not used for data analysis. (DOCX) [file pone.0292474.s002.docx]

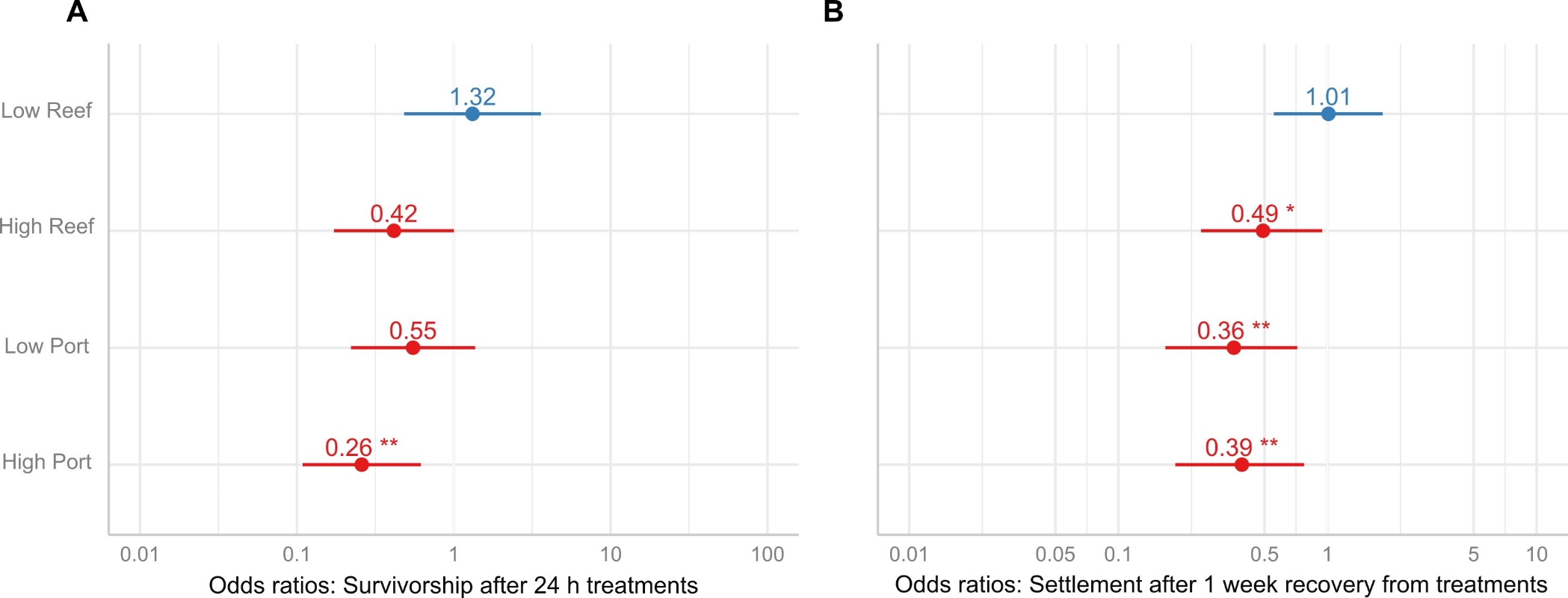

Supplement: S3 Fig — (A) larval survival after 24 h of treatments, and (B) larval settlement after one week of recovery from experimental treatments. OR = 1 Exposure does not affect odds of outcome, OR>1 Exposure associated with higher odds of outcome, and OR<1 Exposure associated with lower odds of outcome. (DOCX) [file pone.0292474.s003.docx]

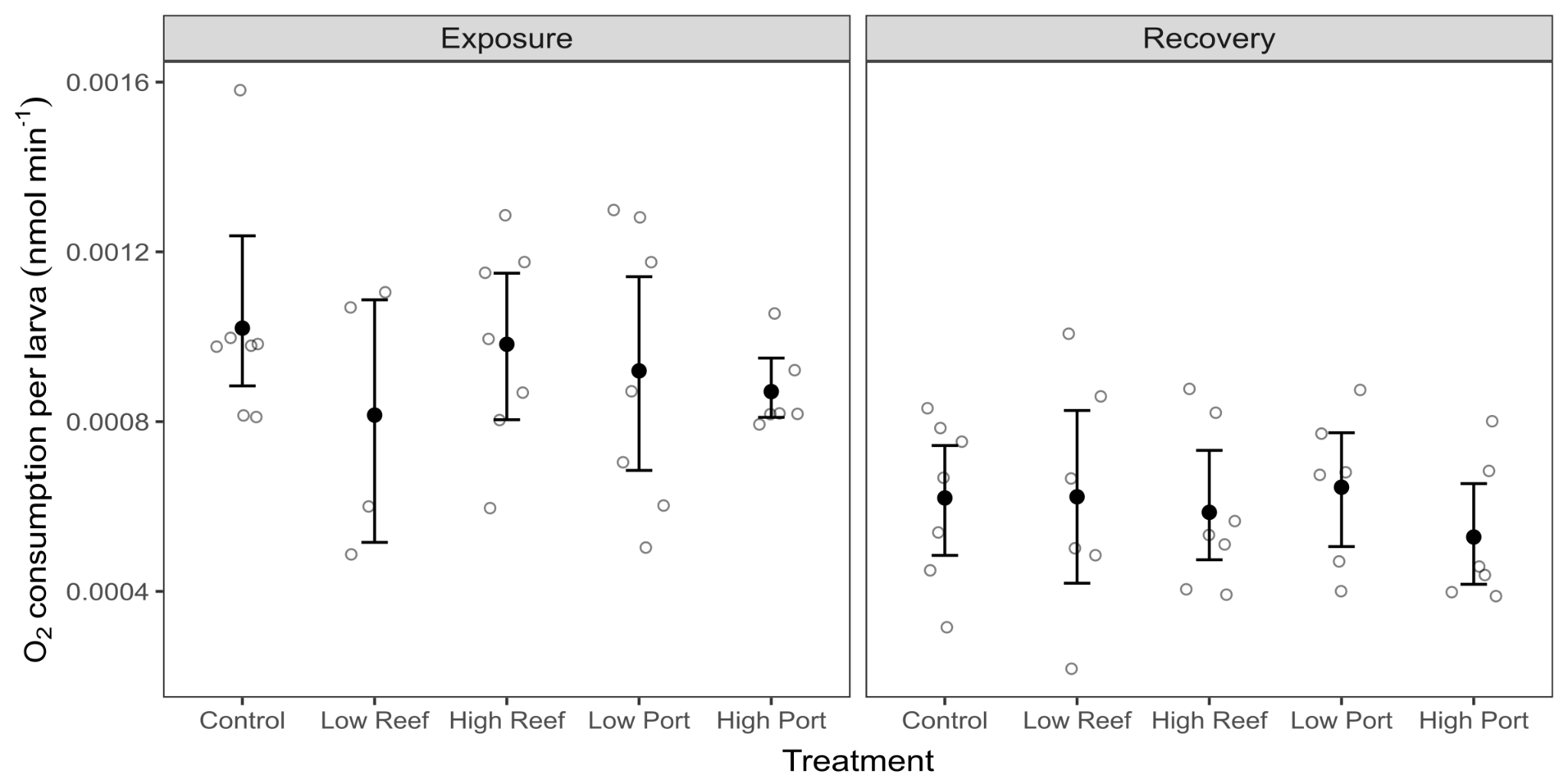

Supplement: S4 Fig — Respiration rates (mean ± 95 CI) in Orbicella faveolata larvae after a 24 h exposure to experimental treatments (left panel) and after one week of recovery from experimental treatments (right panel). White dots denote individual replicate wells. (DOCX) [file pone.0292474.s004.docx]
