# Supplementary material for: Sediment source and dose influence the larval performance of the threatened coral Orbicella faveolata
Source: PLoS One. 2024 Jun 26;19(6):e0292474. doi: 10.1371/journal.pone.0292474 (PMC11207144; doi:10.1371/journal.pone.0292474)
Supplement: S1 Table — Values correspond to graphic in S1 Fig. (DOCX) [file pone.0292474.s005.docx]

| **Sample** | **Port 1-A** | **Port 1-B** | **Reef 1-A** | **Reef 1-B** |
| --- | --- | --- | --- | --- |
| **Graphic Mean** | 1.37 phi  (0.39 mm) | 1.38 phi  (0.38 mm) | 1.0 phi  (0.50 mm) | 0.96 phi  (0.52 mm) |
| **Graphic Standard Deviation**  **(68% of distribution)** | 1.18 phi | 1.13 phi | 0.45 phi | 0.49 phi |
| **Inclusive Graphic**  **Standard Deviation (sorting)** | 1.14 phi  (poorly sorted) | 1.11 phi  (poorly sorted) | 0.49 phi  (well sorted) | 0.50 phi  (well sorted) |
| **Percent mud (<63 μm)** | 1.2 | 1.3 | 0.0 | 0.0 |
| **Percent sand (63 μm – 2 mm)** | 97.4 | 98.1 | 99.7 | 99.7 |
| **Percent gravel**  **(> 2 mm)** | 2.3 | 1.6 | 0.3 | 0.3 |
