# Supplementary material for: Sediment source and dose influence the larval performance of the threatened coral Orbicella faveolata
Source: PLoS One. 2024 Jun 26;19(6):e0292474. doi: 10.1371/journal.pone.0292474 (PMC11207144; doi:10.1371/journal.pone.0292474)
Supplement: S2 Table — npar: Number of parameters, AIC: Akaike information criterion BIC: Bayesian information criterion, logLik: Log likelihood. (DOCX) [file pone.0292474.s006.docx]

| **A) Survival model** | **npar** | **AIC** | **BIC** | **logLik** | **deviance** |
| --- | --- | --- | --- | --- | --- |
| Survivorship ~ Treatment + (1 \| vial) | 6 | 285.38 | 296.48 | -136.69 | 273.38 |
| Survivorship ~ Treatment + (1 \| obs)  (model used) | 6 | 271.36 | 282.46 | -129.68 | 259.36 |
| Survivorship ~ Treatment | 5 | 325.63 | 334.88 | -157.81 | 315.63 |
|  | | | | | |
| **B) Settlement model** | **npar** | **AIC** | **BIC** | **logLik** | **deviance** |
| Settlement ~ Treatment + (1 \| vial) | 6 | 147.64 | 158.35 | -67.822 | 135.64 |
| Settlement ~ Treatment + (1 \| obs) | 6 | 147.64 | 158.35 | -67.822 | 135.64 |
| Settlement ~ Treatment  (model used) | 5 | 145.64 | 154.56 | -67.822 | 135.64 |
