# Supplementary material for: Sediment source and dose influence the larval performance of the threatened coral Orbicella faveolata
Source: PLoS One. 2024 Jun 26;19(6):e0292474. doi: 10.1371/journal.pone.0292474 (PMC11207144; doi:10.1371/journal.pone.0292474)
Supplement: S3 Table — (A) larval survival after 24 h of treatments, and (B) larval settlement after one week of recovery from experimental treatments. The coefficient estimates describe the change in the log odds for each treatment level compared to the base level (control). (DOCX) [file pone.0292474.s007.docx]

|  | 1. **Larval survival** | | | | 1. **Larval settlement** | | | |
| --- | --- | --- | --- | --- | --- | --- | --- | --- |
|  | **Coefficient estimate** | **Std. Error** | **z value** | **Pr(>\|z\|)** | **Coefficient estimate** | **Std. Error** | **z value** | **Pr(>\|z\|)** |
| **Intercept** | 2.8688 | 0.3379 | 8.491 | < 2e-16 *** | -1.2961 | 0.20956 | -6.185 | 6.21e-10 *** |
| **Low Reef** | 0.2757 | 0.5121 | 0.538 | 0.59035 | 0.0109 | 0.30499 | 0.036 | 0.97137 |
| **High Reef** | -0.8783 | 0.4493 | -1.955 | 0.05061 | -1.0311 | 0.30499 | -2.802 | 0.00507 ** |
| **Low Port** | -0.5976 | 0.4651 | -1.285 | 0.19884 | -0.7104 | 0.33885 | -2.096 | 0.03604 * |
| **High Port** | -1.3504 | 0.4436 | -3.045 | 0.00233 ** | -0.9429 | 0.35922 | -2.625 | 0.00867 ** |
