# Supplementary material for: Sediment source and dose influence the larval performance of the threatened coral Orbicella faveolata
Source: PLoS One. 2024 Jun 26;19(6):e0292474. doi: 10.1371/journal.pone.0292474 (PMC11207144; doi:10.1371/journal.pone.0292474)
Supplement: S4 Table — (A) Linear mixed-effect model outputs for Orbicella faveolata larvae respiration. (B) Pairwise comparisons between significant factors. DF: Degrees of freedom, CL: Confidence limit. (DOCX) [file pone.0292474.s008.docx]

| **A) Model outputs** | **Sum Sq** | **Mean Sq** | **DF** | **DenDF** | **F value** | **Pr(>F)** |
| --- | --- | --- | --- | --- | --- | --- |
| Treatment | 7.57e-08 | 1.89e-08 | 4 | 41.18 | 0.63 | 0.64 |
| Timepoint | 4.74e-07 | 4.74e-07 | 1 | 1.94 | 15.83 | 0.06 |
| Treatment:Timepoint | 6.81e-08 | 1.70e-08 | 4 | 33.56 | 0.57 | 0.69 |
|  | | | | | | |
| **B) emmean pairwise comparisons** | | | | | | |
| **Timepoint** | **emmean** | **SE** | **DF** | **Lower CL** | **Upper CL** | **Group** |
| Recovery | 0.000609 | 4.46e-05 | 31.6 | 0.000518 | 0.00070 | 1 |
| Exposure | 0.000928 | 4.55e-05 | 32.5 | 0.000835 | 0.00102 | 2 |
