# Supplementary material for: Sediment source and dose influence the larval performance of the threatened coral Orbicella faveolata
Source: PLoS One. 2024 Jun 26;19(6):e0292474. doi: 10.1371/journal.pone.0292474 (PMC11207144; doi:10.1371/journal.pone.0292474)
Supplement: Attachment — Submitted filename: Response to reviewers.docx [file pone.0292474.s009.docx]

Response to reviews

**Manuscript ID PONE-D-23-30672**

Sediment source and dose influence the larval performance of the threatened coral *Orbicella faveolata*

We would like to thank the Academic Editor and the two reviewers assigned for their constructive comments and suggestions; these greatly improved our manuscript. We have carefully evaluated all comments/suggestions and prepared a revised manuscript in the style and format of the journal PLOS ONE.

***Academic Editor comments***

Comment 1: Please ensure that your manuscript meets PLOS ONE's style requirements, including those for file naming. The PLOS ONE style templates can be found at https://journals.plos.org/plosone/s/file?id=wjVg/PLOSOne_formatting_sample_main_body.pdf and

https://journals.plos.org/plosone/s/file?id=ba62/PLOSOne_formatting_sample_title_authors_affiliations.pdf

**Author’s response:** We have prepared a revised manuscript and reviewed it to ensure that it meets PLOS ONE’s style requirements.

Comment 2: We note that Figure 1 in your submission contains map/satellite images which may be copyrighted. All PLOS content is published under the Creative Commons Attribution License (CC BY 4.0), which means that the manuscript, images, and Supporting Information files will be freely available online, and any third party is permitted to access, download, copy, distribute, and use these materials in anyway, even commercially, with proper attribution. For these reasons, we cannot publish previously copyrighted maps or satellite images created using proprietary data, such as Google software (Google Maps, Street View, and Earth). For more information, see our copyright guidelines: http://journals.plos.org/plosone/s/licenses-and-copyright.

We require you to either (a) present written permission from the copyright holder to publish these figures specifically under the CC BY4.0 license, or (b) remove the figures from your submission:

a. You may seek permission from the original copyright holder of Figure 1 to publish the content specifically under the CC BY 4.0 license.

We recommend that you contact the original copyright holder with the Content Permission Form (http://journals.plos.org/plosone/s/file?id=7c09/content-permission-form.pdf) and the following text:

“I request permission for the open-access journal PLOS ONE to publish XXX under the Creative Commons Attribution License (CCAL) CC BY 4.0 (http://creativecommons.org/licenses/by/4.0/). Please be aware that this license allows unrestricted use and distribution, even commercially, by third parties. Please reply and provide explicit written permission to publish XXX under a CC BY license and complete the attached form.”

Please upload the completed Content Permission Form or other proof of granted permissions as an "Other" file with your submission.

In the figure caption of the copyrighted figure, please include the following text: “Reprinted from [ref] under a CC BY license, with permission from [name of publisher], original copyright [original copyright year].”

b. If you are unable to obtain permission from the original copyright holder to publish these figures under the CC BY 4.0 license or if the copyright holder’s requirements are incompatible with the CC BY 4.0 license, please either i) remove the figure or ii) supply a replacement figure that complies with the CC BY 4.0 license. Please check copyright information on all replacement figures and update the figure caption with source information. If applicable, please specify in the figure caption text when a figure is similar but not identical to the original image and is therefore for illustrative purposes only.

The following resources for replacing copyrighted map figures may be helpful:

USGS National Map Viewer (public domain):

http://viewer.nationalmap.gov/viewer/

The Gateway to Astronaut Photography of Earth (public domain):

http://eol.jsc.nasa.gov/sseop/clickmap/

Maps at the CIA (public domain):

https://www.cia.gov/library/publications/the-world-factbook/index.html and

https://www.cia.gov/library/publications/cia-maps-publications/index.html

NASA Earth Observatory (public domain):

http://earthobservatory.nasa.gov/

Landsat:

http://landsat.visibleearth.nasa.gov/

USGS EROS (Earth Resources Observatory and Science (EROS) Center) (public domain):

http://eros.usgs.gov/#

Natural Earth (public domain):

http://www.naturalearthdata.com/

**Author’s response:** As suggested, figures 1A, 1B, and 1C have been revised using basemaps that comply with the CC BY 4.0 license. Figures 1A and 1C use the World Imagery Base Map from ESRI. Figure 1B uses the USGS ImageryOnly Base Map from the USGS National Map Viewer. The copyright information has now been included in the revised figure caption and the service layer credits are also displayed in each figure. In addition, we removed the words “Miller et al. 2016” from Figure 1C which may have erroneously given the impression that it was a copyrighted figure. Finally, the original figures 1D and 1E were photographs taken by one of the authors but these have been deleted to address one of Reviewer #1 concerns.

Comment 3: Note from Emily Chenette, Editor in Chief of PLOS ONE, and Iain Hrynaszkiewicz, Director of Open Research Solutions at PLOS: Did you know that depositing data in a repository is associated with up to a 25% citation advantage (https://doi.org/10.1371/journal.pone.0230416)? If you’ve not already done so, consider depositing your raw data in a repository to ensure your work is read, appreciated and cited by the largest possible audience. You’ll also earn an Accessible Data icon on your published paper if you deposit your data in any participating repository (https://plos.org/open-science/open-data/#accessible-data).

**Author’s response:** Thank you for the suggestion. All our data and code has been deposited in various repositories recommended by PLOS ONE. All the data and code for the larval survivorship, settlement and oxygen consumption analysis can be found in Zenodo at https://doi.org/10.5281/zenodo.8022069. Raw reads and metadata for the microbial 16S of sediment samples are available in NCBI’s SRA under BioProject ID PRJNA1008169. Finally, all data and code for the sediment microbial analysis can be found at <https://github.com/srosales712/Larvae_Sediment>.

***Reviewer #1 summary***

Serrano et al present a nice study in a well written paper, with interesting results. The strong effect of suspended sediments, particularly from Port Miami, on coral larval performance has wide reaching implications and will make an important contribution to the literature. Before acceptance, I have a couple of potentially major comments that need to be responded to, along with a few minor changes recommended.

***Major comments***

Comment 1: My main comment is in relation to the sediments used and their similarity, particularly looking at Fig 1D:E. The premise of the paper is that the suspension/resuspension of sediments may negatively affect larval performance, but if Fig 1D and E are good representations of the sediments used then they suggest that reef sediments are primarily sand based (E looks like a sand patch) and port sediment laden turf algae (D looks like sediment laden turf on top of hardbottom). This would also explain much of the difference between sediment composition. Both these sediment types are likely present in both locations but you’d expect different responses of larval performance and microbial community to each (coarser sand or finer sediment). It may just be that a more appropriate image is used which shows the sediments used, but if not some justification of why sediment from equivalent habitats (i.e., both sediment laden turf on hardbottom, or both sand patches) was not used.

**Author’s response:** To clarify, the aim of our study was to use sediments representative from these two different sites (Port vs. Reef) to use in our laboratory experiments, not sediments from equivalent habitats within these sites. As stated in the methods section, 3-4 containers with surficial sediments were collected per site and combined to produce a single “batch” for experiments. Thus, the pictures do not show the only area where sediments were collected from at each site. Since we do not have actual pictures of all the areas where the sediments were collected from, for clarity, we have deleted the pictures from Figure 1. Also, note that the grain size analysis conducted for both types of sediment (Port and Reef) showed that these were primarily characterized as sand (>97.4%, see Table S1 and Figure S1), with only trace amounts of mud-sized particles in the Port sediments.

Comment 2: I realise the aim of the study is to assess the impact of dredging activity and resuspension, but some information regarding suspended sediment concentrations expected on natural reefs would be very helpful, particularly as even the low doses (20 mg/L) would be considered high.

**Author’s response:** We appreciate the suggestion. We have now added additional information to the manuscript regarding suspended sediment concentrations typically observed in natural reefs. From our literature search, our low-dose suspended sediment treatments appear to be about twice the concentrations typically observed in natural reefs in southeast Florida (~10 mg/L; Whitall and Bricker, 2021), but within the range observed in inshore reefs in Hawaii (Jokiel et al. 2014), and in turbid reefs in the Great Barrier Reef, which can often exceed 50 mg/L during natural wind-wave events (Larcombe et al. 1995; Cooper et al. 2008; Bessell-Browne et al. 2017).

***Minor comments***

Comment 1: It needs to be clear throughout that the study assesses the impact of suspended sediments (this isn’t mentioned in the abstract for instance).

**Author’s response:** Thank you for your comment; we did not realize that we had not mentioned “suspended sediments” in the abstract. For clarity, we added “suspended sediments” in the abstract and at various other sections throughout the manuscript.

Comment 2: L29 – suggest noting exposed to suspended sediments

**Author’s response:** Done.

Comment 3: L74- 84 - I find this paragraph a bit misleading in relation to the experiment as the influence of benthic sediment on settlement/survival isn’t what's assessed. The information here is fine, but it would benefit from being clear that most of the focus of the effect of sedimentation on recruitment focuses on the benthos and not suspended sediments.

**Author’s response:** Agree. The paragraph was modified to include a short overview of the effects of suspended sediments on coral larval survival and settlement as well.

Comment 4: Suggest moving section on respiration in the methods before the settlement assay section so that the experimental design is chronological.

**Author’s response:** Done.

Comment 5: L213 – how long were respiration experiment runs?

**Author’s response:** The respiration runs varied in length (depending on the larval oxygen consumption), but typically lasted approximately 3-4 hours (Figure S2 shows an example plot over time).

Comment 6: L235 – this is a GLMM not a GLM as you’re using a random effect

**Author’s response:** The sentence was changed to GLMM to specify that the generalized linear mixed model includes a random effect.

Comment 7: L242 - Was model selection performed to assess whether both factors have a significant effect?

**Author’s response:** Yes. We now specify in the text that “Model selection was performed with stepwise backward elimination of non-significant terms using the “step” function of the lmerTest R package”.

Comment 8: L243 - How did you assess post-hoc differences or did you just compare to control for base GLM summary? How were models validated to assure assumptions met?

**Author’s response:** Pairwise differences between the Control and each of the other four treatments were evaluated with the emmeans package with an alpha value of 0.05. This has been added to the manuscript. Treatment was not a significant factor in oxygen consumption, so we did not perform a post-hoc test in this model. Best binomial models were chosen based on the AIC and are now all shown in the S2 Table. The best linear model was chosen by backward elimination of non-significant terms using the “step” function. This was also added to the methods.

Comment 9: L262 - Why did you use euclidean distance to calculate dissimilarity matrix rather than methods more suitable for biotic community data such as Bray-Curtis?

**Author’s response:** We used Euclidean distance because we followed the Compositional Data Analysis (CoDa) method. Bray-Curtis methods transform the data into ratios or relative abundance. CoDA methods move away from this method and transform the data using Centered Log-Ratios (CLR), which are used for compositional data like our sequence data. CLR uses the geometric mean of read counts from all taxa within a sample as the reference or denominator. This method involves dividing all taxon read counts in a sample by this geometric mean, allowing for the comparison of log-fold changes in this ratio between different samples. In this paper, we used Euclidean distance to achieve an Aitchison distance (CLR transformed data on a Euclidean space), which is the recommended distance matrix for compositional data

(https://www.ncbi.nlm.nih.gov/pmc/articles/PMC5695134/).

Comment 10: L273 – 278 - I suggest sticking to the results of the experiments and the statistical tests performed here rather than the semi-qualitative descriptions used, particularly if you’re stating significant impact - Please provide detail on analysis - at minimum the test performed and the p value.

**Author’s response:** As suggested, we removed the qualitative descriptions used in this paragraph and provided the test performed and the p-value.

Comment 11: Fig 2. Caption – please note test used for significance

**Author’s response:** Done. Test was added to the caption.

Comment 12: L305 – please note test significance from

**Author’s response:** It was an ANOVA but this difference was not significant between sites (Fig 3A). For clarity, we added the p-values to the text.

Comment 13: Fig 3 - Should the title in 3A plot 1 be Richness not observed?

**Author’s response:** We believe this is interchangeable (it doesn’t affect the meaning either way) and prefer to leave the figure caption as is.

Comment 14: L359 – 361 - This differs to what Fig 2 says, please check this statement is correct.

**Author’s response:** Thanks for pointing out this error. Based on recommendations from Reviewer #2 we have corrected the data for overdispersion which also changed these results. These changes have now been incorporated into the manuscript.

Comment 15: L371-372 - This doesn't seem to have been statistically tested in the results and Fig 2 suggests no difference between high port and high survivorship.

**Author’s response:** We believe the reviewer meant to write “no difference between high port and high reef survivorship”. Based on recommendations from Reviewer #2 we have corrected the data for overdispersion which also changed these results. These changes have now been incorporated into the manuscript and results from the statistical analysis incorporated in the Results section.

Comment 16: L387 – 390 - Similarly to in the intro, I don't understand why sediment covering on settlement surfaces is discussed as if it may have influenced the results. Maybe I've missed something here but the settlement assays didn't include any sediment but used filtered seawater, so sediment on surfaces is not what's affecting settlement? I think it's interesting enough that you find such strong effects just from suspended sediment.

**Author’s response:** Thank you for your comment. We realized this was an oversight. The introduction and discussion sections were modified to include a short overview of the effects of suspended sediments on coral larval survival and settlement. We also included a few relevant references in these sections as pertinent.

Comment 17: L432 – SCTLD first appearing at Port Miami late isn't entirely true ... see Jones et al 2021 (<https://doi.org/10.1038/s41598-021-93111-0>) or Hayes et al 2022

(doi:10.3389/fmars.2022.975894) for suggestion that SCTLD was present prior to this.

**Author’s response:** Thank you for the comment. We have modified this sentence to clarify that SCTLD was “first reported in outbreak levels (>5% prevalence) in waters of Miami-Dade county around September 2014 (...) but see Jones et al. (2021) and Hayes et al. (2022)”.

***Reviewer #2 summary***

This paper delves into the issue surrounding port development and dredging near Miami, which is a topical and controversial area of research. Predicting how corals may respond in such environments that are naturally already turbid is crucial to understanding the risk associated with these operations. The study investigates effects of suspended sediments on larvae, and latency effects of this exposure on settlement. Sediments-stress experiments are complicated to undertake because no two sediments are the same, and several mechanisms may be at play.

This study did an adequate job at sediment characterisation and measuring the biological responses. Overall, I suggest accepting the manuscript with major revisions. I write ‘major’ because I believe the data analysis needs to be checked, and the results altered to reflect this if changes are made. However, these changes should be reasonably quick to make.

***Major comments:***

Comment 1: The effect sizes here (differences from the control) for larval survivorship are quite modest, even at elevated SSCs. Personally, I would interpret this as larvae survival being initially quite robust to suspended sediment exposure. The settlement data however, shows stronger effects (>50% decrease) indicates a much greater concern despite some uncertainty.

**Author’s response:** Thank you for your feedback and for taking the time to look at the data and code. Based on the corrections suggested in comment #5 (below), the results and discussion have been modified accordingly. The survivorship results now only show significant differences between the Control and the High Port treatment, which we agree, seems to suggest an initial robust response to suspended sediments. However, both Port sediment treatments significantly lowered settlement rates compared to the control treatment, which we also agree presents a much greater concern due to the strong latent effects observed.

Comment 2: Some feedback for future studies would be:

- Use a greater number of treatment levels so that ‘effect thresholds’ are derived rather than NOEC/LOEC thresholds, which are produced from categorical analyses (Warne & van Dam 2008).
- Better experimental justification of the treatment levels. Surprisingly, the authors justify the suspended sediment concentrations from Australian dredging operations, rather than those from the Port of Miami. A quick scan of the grey literature and I was able to find government reports on suspended sediments in the region.
- More testing of the sediment suspending apparatus would have been useful. At the grain size reported, it is likely lots of agitation would be needed to maintain these in suspension and some may have settled out. i.e., responses could be worse than reported because the actual SSC might be lower than what was initially added.

**Author’s response:** Agree these are great suggestions for future studies. We have addressed each of the suggestions below.

- We agree that it would be great to use a larger number of treatment levels in future studies. In our study, since we were working with limited amounts of coral larvae (*O. faveolata* is a threatened species under the Endangered Species Act), our focus was on testing realistic and environmentally-relevant exposure scenarios as it pertains to dredging, given that the two major commercial shipping ports in southeast Florida are currently planning major expansions and this information is lacking (and needed) to complete ESA Section 7 consultations.
- As stated above, our focus was on testing realistic and environmentally-relevant exposure scenarios as it pertains to dredging, and we believe that the work conducted in Australia as part of the Western Australian Marine Science Institution (WAMSI) Dredging Science Node is way more robust than any monitoring compliance reports from the Port of Miami (which only report turbidity values, not total suspended sediment concentrations) for our justification of the treatments. Having said that, we agree that additional information regarding suspended sediments near the study area is warranted in the manuscript, and thus we added an additional reference (Whitall and Bricker, 2021), where the authors found that reef habitats in Southeast Florida had an average TSS concentration of approximately 10 mg/L. This is lower than the lowest treatment we used (~20 mg/L), but within the range observed in inshore reefs in Hawaii (Jokiel et al. 2014), and in turbid reefs in the Great Barrier Reef, which can often exceed 50 mg/L during natural wind-wave events (Larcombe et al. 1995; Cooper et al. 2008; Bessell-Browne et al. 2017). This was also added to the manuscript.
- We conducted multiple trials to assess the best titer shaker settings and exposure times that caused minimal mortality in control larvae while maintaining both fine and coarse sediment particles in continuous suspension. We agree, however, that a small portion of these sediments may have settled out during the exposure period, though it wasn’t anything visually obvious. So yes, it is possible that the responses could be worse than those reported.

Comment 3: Strengths

- For the most part, the experiment has a large sample size, with plenty of replication.
- The paper was written very clearly and was easy to follow. Thank you for that.
- The microbial sediment analysis supplemented the study well, this is not often done and can point to some mechanisms.

**Author’s response:** Thank you so much for these comments. We appreciate them.

Comment 4: Weaknesses

- The study missed two crucial papers on the topic that closely matched the design used here (Ricardo et al. 2016, Humanes et al. 2017). Hopefully this was just an oversight. These studies found no real effects survivorship on larvae following sediment exposure, but some latency effects on settlement at very high SSCs in one study.

**Author’s response:** Thank you so much for this comment, and agree, this was an oversight. We have modified the introduction and discussion sections and incorporated these relevant references as pertinent.

Comment 5: Thank you for providing the data and code. However, even though the approach appears mostly sound, it seems there may be an issue in the statistical analyses for the survival and settlement data. The plots indicate 3 treatment levels are significant but it is clear that this is not the case by looking at the overlapping 95% CI. Reanalysis of the survival data only points to 1 treatment (‘High Port’) level being ‘significant’ compared to the control. This issue may be caused by how observational-level random effects are implemented, and which postdoc test is used (Harrison 2015).

Survival.data1$obs <- factor(seq_len(nrow(Survival.data1))) # unique container/observation ID

fit1<-glmer(cbind(Swimming, Dead) ~Treatment +(1|obs),

data=Survival.data1,family="binomial")

library(emmeans)

contrast(emmeans (fit1, ~ Treatment), "trt.vs.ctrl1", ref = 'Control')

**Author’s response:** Thank you for taking the time to look at the code. We learned something new and have now corrected for overdispersion as needed. The repository code, methods, results, and discussion have been modified accordingly.

Comment 6: Please write the exact p-values rather than <0.05 (unless <0.001)

**Author’s response:** As suggested, we have revised the manuscript text and Fig 2 and included the exact p-values.

***Minor comments***

Comment 1: L108 Gamete ‘egg-sperm’

**Author’s response:** Done. We added the suggested words.

Comment 2: L117 – How were the larvae transported?

**Author’s response:** Larvae was transported in bins with lids and aeration. This was added to the manuscript.

Comment 3: L143 - How long was this? Was there any water exchange?

**Author’s response:** As stated in the methods section, the two batches of sediments were collected 24-48 hrs before the start of the experiment. No water exchange was done after sediments were combined into a single “batch” in the laboratory, but the sediments were continuously moved and kept aerated during this period.

Comment 4: L144 Can you explain the wet weigh to dry weight procedure a bit more.

**Author’s response:** Aliquots of the wet sediments were oven dried in the laboratory for analysis of grain size (as described in the methods section) and calculation of dry weight. The wet weights were then adjusted for the percent moisture content in the sediments prior to preparing the different treatments. This information has been added to the manuscript.

Comment 5: L235 generalized linear ‘mixed ‘models (‘GLMM’)

**Author’s response:** Yes, thank you. “Mixed” has been added to the model description and acronym**.**

Comment 6: L302 – Please explain which sequencing filtering steps were taken.

**Author’s response:** The steps for filtering were previously described in the methods section where it said “Alpha-diversity was evaluated by rarefying to a minimum sequence depth of 60,000”. We have now also added that we removed the Amplicon Sequence Variants (ASVs) that had zero across all samples to add more details to this step.

Comment 7: L341-342 This sentence is probably better suited to the Methods

**Author’s response:** As suggested, this sentence was moved to the methods section.

Comment 8: I was initially concerned that conducting a settlement assay on a random piece of rubble would yield no settlement. Corals are selective about settlement substrates and *O. faveolata* has been shown to show preferences to types of CCA. So I relieved to see settlement at ~20% in the control, which is typical of this species, but much lower than many Indo-pacific corals. That said, I think it is worthwhile in the Discussion or the Methods outlining that *O. faveolata* typically shows very low settlement success and providing a few references. As an aside, I do wonder if the preferred settlement substrate for *O. faveolata* has not been found, or if the settlement peak is very protracted.

**Author’s response:** Thank you for this observation. We have regularly used ambient reef rubble similar to this study for a 'positive control' for settlement experiments and have often gotten mean settlement of over 40% (in a 24 hr period) for this species in this way. It does vary by year, and for example, Miller et al. 2020 reported a maximum settlement of about ~20% in 2014. This information has now been included in the discussion section of the revised manuscript.

Comment 9: One point to consider is that even though 100 mg/L is considered very high (it is closest to the 100% percentile in US EPA MOMSreport), larvae move passively within a plume and therefore might have a different exposure time to an in situ nephelometers from where most WQ data come from.

**Author’s response:** Yes, we agree. Thank you for the comment. It is concerning though that we saw significant latent effects on settlement with just a 24 hr exposure to a low dose of Port suspended sediments, which suggest that even brief exposures to these sediments through passive transport could be detrimental to key biological processes for this species.

Comment 10: L368 – Did you notice any mucous production from the larvae from clearing?

**Author’s response:** No we didn’t see any mucous production. Having said that, our exposure period was shorter than that from Ricardo et al. 2016 (60 hrs) in which the authors observed ciliated larvae secreting mucus, so it is possible that it takes a longer exposure period for *O. faveolata* larvae to secrete mucus as well. Alternatively, the lack of mucus production (and thus lack of this protective mechanism) may help explain why we observed significant effects at much lower suspended sediment concentrations compared to Ricardo et al. 2016. This has now been included as part of the discussion in the revised manuscript.

Comment 11: L386 – This reference/hypothesis is unclear. Please cite the actual study rather than the literature review

**Author’s response:** Done. We have modified this sentence and cited the appropriate study.

Comment 12: L393. There have been other studies that have done this (see earlier).

**Author’s response:** Thanks for pointing this out. We have modified this paragraph and removed this portion of the sentence.

Comment 13: L406. Possibly, but Humanes 2017 uses wet sediments and did not find latency effects (although they did with pre-exposed embryos).

**Author’s response:** We modified the discussion section to make the clarification that this was not the case in Humanes et al. 2017, and also modified the introduction to make it clear that this is not the first study to use “wet” sediments.

Comment 14: L400. ‘we speculate that biological/chemical damage to larvae exposed to Port sediments may have disrupted the detection of settlement cues’ is maybe clearer.

**Author’s response**: Agree. Thank you for the suggestion. We have modified the sentence as suggested.
