# Supplementary material for: Sediment source and dose influence the larval performance of the threatened coral Orbicella faveolata
Source: PLoS One. 2024 Jun 26;19(6):e0292474. doi: 10.1371/journal.pone.0292474 (PMC11207144; doi:10.1371/journal.pone.0292474)
Supplement: Attachment — Submitted filename: Response to reviewers R1.docx [file pone.0292474.s010.docx]

Response to reviews

**Manuscript ID PONE-D-23-30672R1**

Sediment source and dose influence the larval performance of the threatened coral *Orbicella faveolata*

We have carefully evaluated the minor comments and suggestions received and prepared a revised manuscript in the style and format of the journal PLOS ONE.

***Academic Editor comments***

Comment 1: Please review your reference list to ensure that it is complete and correct. If you have cited papers that have been retracted, please include the rationale for doing so in the manuscript text, or remove these references and replace them with relevant current references. Any changes to the reference list should be mentioned in the rebuttal letter that accompanies your revised manuscript. If you need to cite a retracted article, indicate the article’s retracted status in the References list and also include a citation and full reference for the retraction notice.

**Author’s response:** We have reviewed our reference list to ensure that is complete and correct. We also used the style file (PLoS) offered by EndNote to assist with formatting our references. Note that no changes to the reference list were made in this revised version of the manuscript (PONE-D-23-30672R1). However, below we provide a list of all the references that were replaced, added or removed in our previous version of the manuscript and rationale for doing so.

1) We removed one reference (Vazquez, 2023, citation below) and replaced it with the citation of the actual Port Miami report from NOAA referenced in the Local 10 News.

Vazquez C. Explosive report finds PortMiami dredging caused extensive coral reef damage. Local 10 News [Internet]. 2023 Sept 06. Available from:

<https://www.local10.com/news/local/2023/09/06/explosive-report-finds-portmiami-dredging-caused-extensive-coral-reef-damage/#:~:text=MIAMI%20%E2%80%93%20A%20new%20federal%20report,along%20the%20port's%20entrance%20channel>.

Replaced with:

[45] Karazsia J, Mack K, Wilber P, Miller M, Griffin S, Moore T. Examination of Sedimentation Impacts to Coral Reef along the Port Miami Entrance Channel, December 2015 and April 2016. Available from:

https://www.ncei.noaa.gov/data/oceans/coris/library/NOAA/CRCP/NMFS/SERO/Projects/30049/Karazsia2023_PortMiami_Dredge_PhaseIII_ImpactAssessment_FinalReport.pdf: 2023.

2) We added several references in our previous version of the manuscript to address the two reviewers’ concerns and suggestions. Most of these references were identified in our previous rebuttal letter and all references were shown as tracked changes. These references were incorporated in the R1 manuscript and correspond to the following citations:

[25] Gilmour J. Experimental investigation into the effects of suspended sediment on fertilisation, larval survival and settlement in a scleractinian coral. Marine Biology. 1999;135:451-62.

[26] Humanes A, Ricardo GF, Willis BL, Fabricius KE, Negri AP. Cumulative effects of suspended sediments, organic nutrients and temperature stress on early life history stages of the coral Acropora tenuis. Scientific Reports. 2017;7(1):44101.

[43] Ricardo GF, Jones RJ, Clode PL, Negri AP. Mucous secretion and cilia beating defend developing coral larvae from suspended sediments. PLoS One. 2016;11(9):e0162743.

[51] Whitall D, Bricker S. Examining Ambient Turbidity and Total Suspended Solids Data in South Florida Towards Development of Coral Specific Water Quality Criteria. 2021. doi:

doi.org/10.25923/v35e-cv79.

[52] Jokiel PL, Rodgers KuS, Storlazzi CD, Field ME, Lager CV, Lager D. Response of reef corals on a fringing reef flat to elevated suspended-sediment concentrations: Molokai, Hawaii. PeerJ. 2014;2:e699.

[54] Cooper TF, Ridd PV, Ulstrup KE, Humphrey C, Slivkoff M, Fabricius KE. Temporal dynamics in coral bioindicators for water quality on coastal coral reefs of the Great Barrier Reef. Marine and Freshwater Research. 2008;59(8):703-16.

[55] Larcombe P, Ridd P, Prytz A, Wilson B. Factors controlling suspended sediment on inner-shelf coral reefs, Townsville, Australia. Coral reefs. 1995;14:163-71.

[61] Harrison XA. A comparison of observation-level random effect and Beta-Binomial models for modelling overdispersion in Binomial data in ecology & evolution. PeerJ. 2015;3:e1114.

[63] Searle SR, Speed FM, Milliken GA. Population marginal means in the linear model: an alternative to least squares means. The American Statistician. 1980;34(4):216-21.

[70] Miller MW, Bright AJ, Pausch RE, Williams DE. Larval longevity and competency patterns of Caribbean reef-building corals. PeerJ. 2020;8:e9705.

[78] Jones NP, Kabay L, Semon Lunz K, Gilliam DS. Temperature stress and disease drives the extirpation of the threatened pillar coral, Dendrogyra cylindrus, in southeast Florida. Scientific Reports. 2021;11(1):14113.

[79] Hayes NK, Walton CJ, Gilliam DS. Tissue loss disease outbreak significantly alters the Southeast Florida stony coral assemblage. Frontiers in Marine Science. 2022;9. doi:

doi.org/10.3389/fmars.2022.975894.

[82] Riegl B, Branch GM. Effects of sediment on the energy budgets of four scleractinian (Bourne 1900) and five alcyonacean (Lamouroux 1816) corals. Journal of Experimental Marine Biology and Ecology. 1995;186(2):259-75.

[83] Telesnicki G, Goldberg W. Effects of turbidity on the photosynthesis and respiration of two south Florida reef coral species. Oceanographic Literature Review. 1996;2(43):199.

3) Two references were removed from our previous version of the manuscript to help focus our introduction on the effects of suspended sediments on larval settlement and address the suggestions provided by reviewer #1. The citations for these references are below:

Price N. Habitat selection, facilitation, and biotic settlement cues affect distribution and performance of coral recruits in French Polynesia. Oecologia. 2010;163(3):747-58. Epub 2010/02/20. doi:

10.1007/s00442-010-1578-4.

Ritson-Williams R, Paul VJ, Arnold SN, Steneck RS. Larval settlement preferences and post-settlement survival of the threatened Caribbean corals Acropora palmata and A. cervicornis. Coral Reefs. 2009;29(1):71-81. doi: 10.1007/s00338-009-0555-z.

***Reviewer #1 summary***

Authors appropriately answered all comments and I recommend publishing the manuscript. There are a couple of minor things I noticed that the authors may want to check.

Comment 1: One minor typo in table S2b - settlement should be the response in all model equations.

**Author’s response:** Thanks for noticing the error. We have corrected it.

Comment 2: Also, check p-values in text L297-308 - I presume the values stated in text are from the emmeans pairwise comparisons and not the glmm summary that’s in table S3? I got confused at first because S3 table is referenced.

**Author’s response:** Yes, that is correct. We have modified the text in lines 297-308 to indicate that the p-values refer to figure 2A and 2B and not the results from the glmm summary that is in table S3.

***Reviewer #2 summary***

The authors have adequately addressed all of my comments and concerns and I look forward to seeing this manuscript published.
